# Effects of intra-arterial transplantation of adipose-derived stem cells on the expression of netrin-1 and its receptor DCC in the peri-infarct cortex after experimental stroke

**DOI:** 10.1186/s13287-017-0671-6

**Published:** 2017-10-10

**Authors:** Huan Huang, Fan Lin, Jingjing Jiang, Yan Chen, Ainong Mei, Pengli Zhu

**Affiliations:** 1 Department of Geriatric Medicine, Fujian Provincial Hospital, 134 Dongjie Road, Fuzhou, Fujian 350001 China; 2 Provincial Clinical Medical College of Fujian Medical University, 134 Dongjie Road, Fuzhou, Fujian 350001 China; 3 Fujian Key Laboratory of Geriatrics, 134 Dongjie Road, Fuzhou, Fujian 350001 China

**Keywords:** Stroke, Adipose-derived stem cells, Netrin-1, DCC, Regeneration, Neurological recovery

## Abstract

**Background:**

Stem cell transplantation has been documented to promote functional recovery in animal models of stroke; however, the underlying mechanisms are not yet fully understood. As netrin-1 and its receptor deleted in colorectal cancer (DCC) are important regulators in neuronal and vascular activities, the present study attempted to explore whether netrin-1 and DCC are involved in the neuroprotection of stem cell-based therapies in a rat ischemic stroke model.

**Methods:**

Adult male Sprague–Dawley rats were subjected to a transient middle cerebral artery occlusion (MCAO) and subsequently received an intra-arterial injection of 2 × 10^6^ PKH26-labeled adipose-derived stem cells (ADSCs) or saline 24 h later. Neurological function was evaluated by behavioral tests before the rats were sacrificed at days 7 and 14 after MCAO. The migration of ADSCs and regeneration of neuronal fibers and blood vessels were determined by immunofluorescence staining. The expression of netrin-1 and DCC was analyzed by Western blot and immunofluorescence staining.

**Results:**

ADSC transplantation significantly improved the neurological recovery at days 7 and 14, and noticeably promoted the regeneration of neuronal fibers and blood vessels in the peri-infarct cortex at day 14. PKH26-labeled ADSCs located mainly in the peri-infarct area at days 7 and 14. In ADSC-treated rats, the expression of netrin-1 and DCC significantly increased in the peri-infarct cortex at days 7 and 14. Immunofluorescence staining showed that netrin-1 was mainly expressed by neuronal perikaryal in the peri-infarct cortex, and DCC was mainly expressed by neuronal fibers and was present around the blood vessels in the peri-infarct cortex.

**Conclusions:**

These findings suggest that ADSC transplantation facilitates the regeneration of neuronal fibers and blood vessels in the peri-infarct cortex and improves neurological functions, which may be attributed, at least in part, to the involvement of upregulated netrin-1 and DCC in the remodeling of neuronal and vascular networks in the peri-infarct cortex.

## Background

Ischemic stroke continues to be a major cause of death and disability, imposing a mounting burden on individuals, households, and society as a whole [1, 2]. So far, only intravenous recombinant tissue plasminogen activator (rtPA) is approved for the treatment of patients afflicted with ischemic stroke. Unfortunately, this treatment scheme has a narrow therapeutic window (within 4.5 h after the symptomatic onset) and other strict eligibility criteria which have largely limited its clinical application. Therefore, few stroke patients can benefit from it and an alternative treatment that can be widely applied is badly needed [2, 3].

Accordingly, stem cell-based therapies have emerged as a promising strategy for the treatment of ischemic stroke [4, 5]. Among various stem cell types, adipose-derived stem cells (ADSCs) present several advantages and can serve as good candidates for cell therapy after stroke [6]. For example, they are derived from adipose tissues and thus are abundant and easy to obtain [7]. Moreover, due to their relatively low immunogenicity, they are able to proliferate and differentiate without producing adverse side effects [4, 8]. Additionally, they can be administered without ethical concerns [9]. Evidence from experimental animal models confirms that ADSC therapy can enhance functional recovery after ischemic stroke [7, 10, 11]. However, the mechanisms of its protective effects are not yet fully understood and await further investigation [5].

As the first axonal guidance cue identified in vertebrates, netrin-1 is primarily known as a strong chemotropic factor for axonal pathfinding activities during neural development [12, 13]. It is now well-established that netrin-1 elicits chemoattractant guidance by binding to its canonical receptors deleted in colorectal cancer (DCC) and neogenin, or chemorepellant guidance by binding to the DCC/uncoordinated (Unc5) A–D receptor complex [14, 15]. Other studies have reported that netrin-1 and its receptors have been engaged in regulating diverse processes, including axonal outgrowth and branching [16], neuronal survival [17, 18] and migration [19], developmental and therapeutic angiogenesis [20], and so on [15, 21]. It has also been shown that netrin-1 and its receptors are highly expressed in the central nervous system during embryonic development and are continuously expressed in the adult central nervous system [22, 23]. In recent years, accumulating evidence indicates that netrin-1 plays a positive role in nerve regeneration and functional recovery [23]. Other studies of animal models have demonstrated that the expression levels of netrin-1 and its receptors (DCC and neogenin) are upregulated after brain ischemia [24, 25], and that intracerebral injection of netrin-1 reduces the number of dying neurons and protects the infarct tissue from p53-mediated apoptosis after stroke [22]. In addition, netrin-1 and its receptors DCC and Unc5B have been demonstrated to be involved in the exercise-induced functional recovery of rats after stroke [25]. Taken together, we hypothesize that netrin-1 and its receptor DCC may also be involved in the beneficial effects of ADSC transplantation on the neurological recovery after ischemic stroke in rats.

To verify this hypothesis and elucidate the underlying mechanisms, the present study tested the effect of ADSC therapy on improving neurological recovery in a rat model of a transient middle cerebral artery occlusion (MCAO). We detected the migration of ADSCs in rat brains after intra-arterial transplantation and further examined the regeneration of neuronal fibers and blood vessels, and the temporal and spatial expression patterns of netrin-1 and DCC in the peri-infarct cortex after ADSC transplantation.

## Methods

### Experimental animals

A total of 82 healthy male Sprague–Dawley rats were used in this study, of which 72 rats (weight 250–280 g) were used for establishing the MCAO model. These rats were randomly divided into four groups: Sham group (*n* = 18), MCAO group (*n* = 18), MCAO + vehicle group (*n* = 18), and MCAO + ADSC group (*n* = 18). The other 10 rats (weight 80–100 g) were used for isolating ADSCs.

### Isolation, culture, and identification of ADSCs

ADSCs were isolated from rats weighing 80–100 g and were cultured primarily as previously described [26]. In brief, the rats were initially anesthetized with isoflurane (3% induction, 1.5% maintenance in 30% O_2_, and 70% N_2_O). Inguinal fat pads of rats were carefully dissected and excised, and then washed in serial dilutions of betadine. The fat tissue was cut into pieces (<1 mm^3^ in size) in phosphate-buffered saline (PBS) with surgical scissors. The homogenized adipose tissues were digested with 0.3% Type I collagenase (Sigma-Aldrich, St. Louis, MO, USA) at 37 °C for 60 min. The digested tissue was filtered through a 100-mesh filter to remove debris, and the filtrate was centrifuged at 1000 rpm for 10 min. The cellular pellet was resuspended and then cultured in Dulbecco’s modified Eagle’s medium (Gibco, Grand Island, NY, USA), which was supplemented with 10% fetal bovine serum (Gibco), 100 U/ml penicillin (Gibco), and 100 g/ml streptomycin (Gibco) in a humidified atmosphere of 95% air and 5% CO_2_ at 37 °C for 24 h. Unattached cells and debris were then removed and the adherent cells were cultured with fresh medium. After reaching a confluency of 80%, cells were released with 0.05% trypsin (Sigma-Aldrich) and then subcultured. Passages up to 3–4 were used for experiments. Cellular characteristics of ADSCs were identified by immunofluorecence staining before transplantation. Cells of passage 3–4 were inoculated in 24-well cell culture plates and fixed with 4% paraformaldehyde for 1 h. Afterwards, the cells were washed with PBS three times and blocked with 3% normal donkey serum (Jackson Immunoresearch, West Grove, PA, USA) for 20 min. The cells were then incubated with primary mouse antibodies against CD34, CD44, and CD45 (1:100, Santa Cruz Biotechnology, Santa Cruz, CA, USA) for 1 h. After washing with PBS three times, cells were incubated with Dylight488 affinipure donkey anti-mouse immunoglobulin G (IgG; 1:400, Jackson Immunoresearch) for 45 min. Finally, the cells were visualized under a fluorescence microscope (Eclipse 80i, Nikon, Tokyo, Japan).

### MCAO procedures

Transient MCAO was induced by establishing an intraluminal vascular occlusion as previously described [27]. Briefly, rats were anesthetized with isoflurane (4% induction, 2% maintenance in 30% O_2_, and 70% N_2_O). The common carotid artery (CCA), external carotid artery (ECA), and internal carotid artery (ICA) on the right side of the rats were exposed through a midline neck incision. A 3.0 monofilament nylon suture with its tip rounded by heating near a flame was introduced from the ECA into the ICA until it blocked the origin of the middle cerebral artery. After 1.5 h of MCAO, reperfusion was achieved by withdrawal of the suture until the tip cleared the lumen of the ICA and reached the origin of ECA. During the surgical procedure, the body temperature of rats was maintained at 37.0 ± 0.5 °C using a heating pad and heating lamp. Rats of the Sham group were subjected to the same surgical procedures as described above except that the monofilament nylon suture was not inserted into the ICA.

### ADSC transplantation

Intra-arterial transplantation of ADSCs was initiated at 24 h after MCAO. Before transplantation, ADSCs were labeled with PKH26 (Sigma) as previously described [28]. Immediately after the insertion of the polyethylene tube (PE-50, Becton, Dickinson and Company, Sparks, MD, USA) into the ICA of rats in the MCAO + ADSC group, approximately 2 × 10^6^ cells suspended in 50 μl of saline were carefully infused through the tube for 10 min, while the blood flow of the CCA was temporally blocked with a microvascular clip. Rats in the MCAO + vehicle group underwent the same procedure but received 50 μl of saline alone. After infusion, the injection site was carefully compressed with Gelfoam (Pfizer, New York, NY, USA) for 10 min. The vital signs of all rats were stable, and no profound bleeding occurred during the procedure. Immunosuppressants were not used in any rat in this study.

### Behavioral tests

All rats were able to perform the behavioral tests and did not exhibit substantial asymmetries before MCAO. At days 7 and 14 after MCAO, all rats underwent behavioral tests by an investigator who was blinded to the treatment groups (*n* = 9 at each time point). The modified neurologic severity scores (mNSS), a composite of the motor (muscle status and abnormal movement), sensory (visual, tactile, and proprioceptive), reflex (pinna, corneal, startle), and balance tests [29], were used to measure various aspects of neurological function on a scale of 0 to 18 (normal score, 0; maximal deficit score, 18), where 1 point is awarded for the inability to perform the test or for a lack of a tested reflex. Accordingly, a higher score indicates more severe neurological injury. All rats were sacrificed successively at each time point after the completion of the above tests.

### Western blot

After rats were sacrificed, the brain tissue corresponding to the peri-ischemic cortex (as described in Fig. 4a) was immediately dissected for Western blot analysis (*n* = 4 at each time point). An equal amount of protein extracts (30 μg) was loaded and separated by sodium dodecyl sulfate–polyacrylamide gel electrophoresis using 8% polyacrylamide gel. After electrophoresis, the separated proteins were transferred electrophoretically to a polyvinylidene difluoride membrane (Millipore, Bedford, MA, USA). The membranes were then immersed in a blocking buffer (5% nonfat milk, 0.1% Tween 20 in tris-buffered saline) at room temperature for 2 h to prevent nonspecific binding. The membranes were subsequently incubated with the following primary antibodies at 4 °C overnight: goat anti-netrin-1 antibody (1:800, Santa Cruz Biotechnology), goat anti-DCC antibody (1:800, Santa Cruz Biotechnology), and mouse anti-GAPDH antibody (1:800, Zhongshan Goldenbridge Biotechnology, Beijing, China). After washing with PBS three times, the membranes were then incubated with appropriate secondary antibodies at room temperature for 1 h: peroxidase-conjugated affinipure rabbit anti-goat IgG (1:8000, Zhongshan Goldenbridge Biotechnology) and peroxidase-conjugated affinipure goat anti-mouse IgG (1:8000, Zhongshan Goldenbridge Biotechnology). The signal was then detected by immersing the membranes in enhanced chemiluminescence solution (Beyotime, Jiangsu, China) and developed on films (Kodak, Xiamen, China). The films were then scanned with a scanner, and the intensity of each netrin-1, DCC and GAPDH band was measured by Image J software (US National Institute of Health, Bethesda, MD, USA). The level of protein in each sample was normalized to its corresponding GAPDH level. The band at day 7 in the Sham group was the calibrator sample representing the 1× expression of each protein.

### Immunofluorescence staining

For the immunofluorescence staining, 6-μm coronal sections of brain were cut with a cryostat (CM1850, Leica Microsystems GmbH, Wetzlar, Germany) and collected on glass slides as previously described (*n* = 5 at each time point) [25]. Some sections from the MCAO + ADSC group were mounted and examined under a fluorescence microscope directly to detect the immigration of PHK26-labeled ADSCs in rat brains at days 7 and 14 after MCAO. The other sections were processed in the following steps to localize the expression patterns of netrin-1 and DCC in rat brains of each group. The sections were washed in PBS, blocked in 3% normal donkey serum and 0.1% Triton X-100 (Amresco, Solon, OH, USA), and then incubated with the following primary antibodies at 4 °C overnight: goat anti-netrin-1 antibody (1:100, Santa Cruz Biotechnology) and goat anti-DCC antibody (1:100, Santa Cruz Biotechnology). To colocalize the expression of the above proteins with specific cellular makers, the following primary antibodies were used: mouse anti-neurofilament-200 (NF-200; 1:100, Boster Biological Technology, Wuhan, China) for neuronal fibers, mouse anti-neuron-specific enolase (NSE; 1:100, Zhongshan Goldenbridge Biotechnology) for neuronal perikarya, mouse anti-von Willebrand factor (vWF; 1:100, Zhongshan Goldenbridge Biotechnology) for blood vessels, and mouse anti-glial fibrillary acidic protein (GFAP; 1:100, Beyotime) for astrocytes. After washing with PBS three times, appropriate secondary antibodies were applied at room temperature for 2 h: Dylight649 affinipure donkey anti-goat IgG and Dylight488 affinipure donkey anti-mouse IgG (respectively, 1:400, Jackson Immunoresearch). Sections were then stained with 4,6-diamidino-2-phenylindole (DAPI) (1:500, Sigma) to identify the nucleus. Negative controls were tested simultaneously with the omission of the primary antibodies. Finally, sections were mounted and examined under a fluorescence microscope, and attention was mainly focused on the expression patterns of interested proteins in the peri-ischemic cortex (as indicated in Fig. 4a).

To determine the effects of ADSC transplantation on the regeneration of neuronal fibers and blood vessels, immunofluorescence staining was also used to measure NF-200^+^ neuronal fibers and vWF^+^ blood vessels in the peri-infarct cortex at day 14 after MCAO as previously described (*n* = 3 for each group) [30]. Every ten sections between Bregma levels + 1.60 and 20.2 mm were selected (total = three sections per brain). Three areas of interest were selected to obtain an average of the density of NF-200^+^ neuronal fibers and the number of vWF^+^ blood vessels in the peri-infarct cortex for each section. An average for the three sections per hemisphere was then calculated. The quantification of the density of NF-200^+^ neuronal fibers and the number of vWF^+^ blood vessels within each fixed field of view was performed using Image-Pro Plus 6.0 software (Media Cybernetics, Bethesda, MD, USA). Data of the density of NF-200^+^ neuronal fibers are presented as a percentage of the Sham group, and the number of vWF^+^ blood vessels is presented as counts per square millimeter. All image analyses were conducted by independent observers who were blinded to the treatment groups.

### Statistical analysis

The statistical software package SPSS 21.0 (IBM Corporation, Armonk, NY, USA) was used for data analysis. All data are presented as mean ± SEM. A one-way analysis of variance followed by either the Bonferroni or Dunnett T3 test was used for multiple group comparisons. The value of *p* < 0.05 was considered statistically significant.

## Results

### Characteristics of cultured ADSCs

**Fig. 1:**
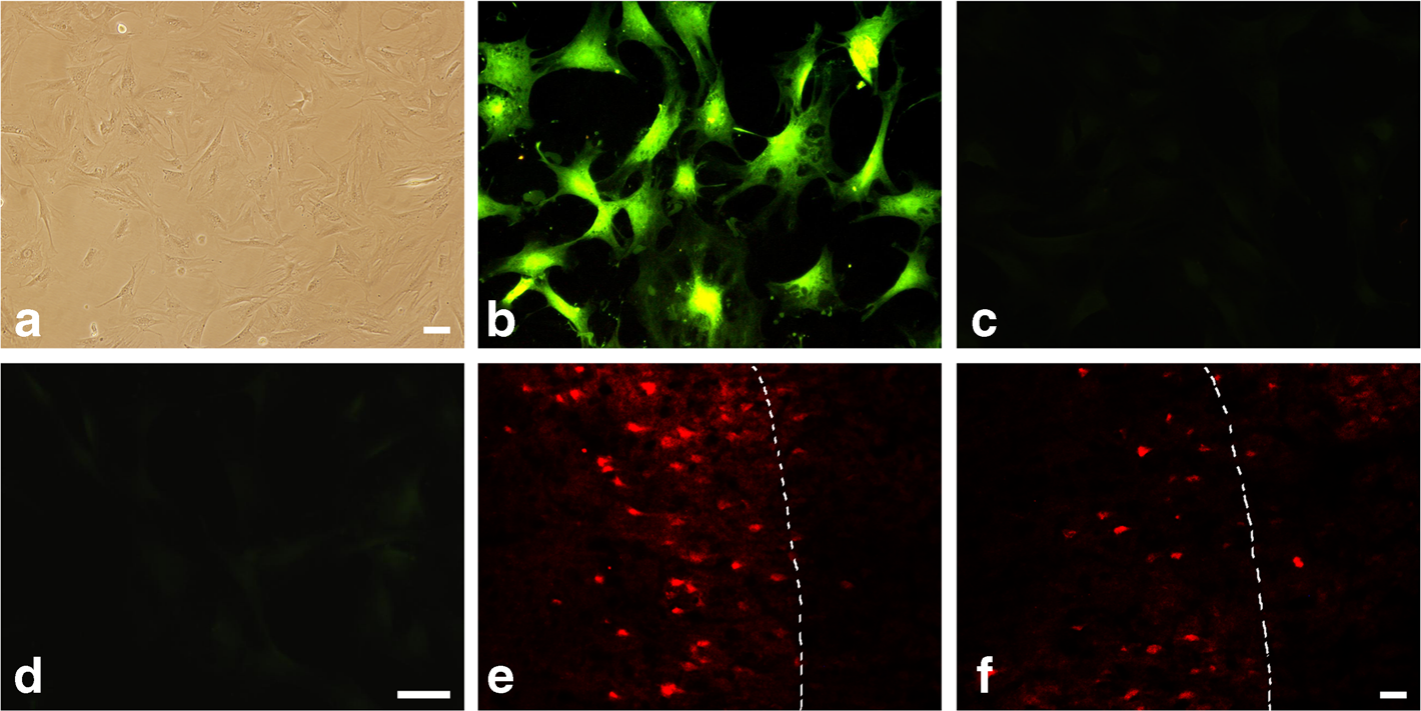
Characteristics and immigration of ADCSs after transplantation. **a** Passage 3 of cultured ADSCs. *Scale bar* = 50 μm. Immunofluorescence staining showed that ADSCs at passages 3–4 were positive for surface antigens CD44 (**b**), while negative for CD34 (**c**) and CD45 (**d**). *Scale bar* = 50 μm. Immunofluorescence staining showed that PKH-26 (*red*)-labeled ADSCs were mainly located in the peri-infarct area at days 7 (**e**) and 14 (**f**) after MCAO, and the number of PKH-26^+^ cells decreased at day 14 when compared to day 7. The *white dotted line* represents the boundary line between the peri-infarct area (left side) and infarct core (right side). *Scale bar* = 50 μm

ADSCs adhered to the bottom and scattered in a number of colony distributions 3 days after plating. At days 12–14 of culture, ADSCs reached a confluency of 80–90% and exhibited a long-spindle form. Passaged cells were uniformly distributed, and covered the bottom every 3–4 days. Within 3–4 passages after the primary culture, ADSCs appeared as a monolayer of large and flat cells. As the cells reached confluency, they exhibited a more spindle-shaped, fibroblastic morphology (Fig. 1a). Immunofluorescence staining showed that ADSCs at passages 3–4 were positive for surface antigens CD44 (Fig. 1b) and negative for CD34 (Fig. 1c) and CD45 (Fig. 1d), which is consistent with previous reports [7].

### Immigration of ADCSs after transplantation

The ADSCs were labeled with PHK-26 before intra-arterial transplantation in order to trace the cells in the brain. Consistent with a previous study [31], at day 7 after MCAO PKH-26^+^ cells (red), indicating the presence of transplanted and migrated ADSCs, were mainly observed in the peri-infarct area (Fig. 1e) while few cells were observed in the infarct core and other cerebral areas. The number of PKH-26^+^ cells decreased at day 14 after MCAO, and the labeled cells were still located mainly in the peri-infarct area (Fig. 1f).

### Behavioral tests

**Fig. 2:**
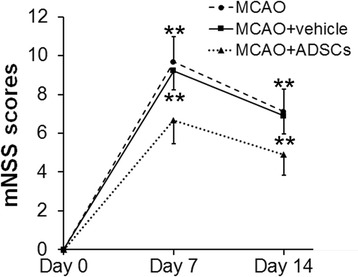
Effect of ADSC transplantation on behavioral tests (mNSS). Compared with rats in the middle cerebral artery occlusion (*MCAO*) group and the MCAO + vehicle group, rats in the MCAO + adipose-derived stem cell (*ADSC*) group showed significantly lower modified neurological severity scores (*mNSS*) at days 7 and 14 after MCAO. **p < 0.01, compared with the MCAO + ADSC group at the corresponding time points

The mNSS was used to assess the effect of ADSC transplantation on the improvement in damaged neurological function after MCAO. The neurologic scores of rats in each group are summarized in Fig. 2. Rats in the MCAO + ADSC group had significantly lower mNSS scores when compared with the MCAO group and the MCAO + vehicle group at day 7 (6.67 ± 1.22 vs. 9.67 ± 1.32 and 6.67 ± 1.22 vs. 9.22 ± 0.97, respectively; *p* < 0.01 for both) and day 14 (4.89 ± 1.05 vs. 7.11 ± 1.17 and 4.89 ± 1.05 vs. 6.89 ± 0.93, respectively; *p* < 0.01 for both). No significant difference was found between the MCAO group and the MCAO + vehicle group at each time point (*p* > 0.05). The results suggest that ADSC transplantation significantly improves the neurological recovery in the acute phase of stroke in rats.

### Regeneration of neuronal fibers in the peri-infarct cortex

**Fig. 3:**
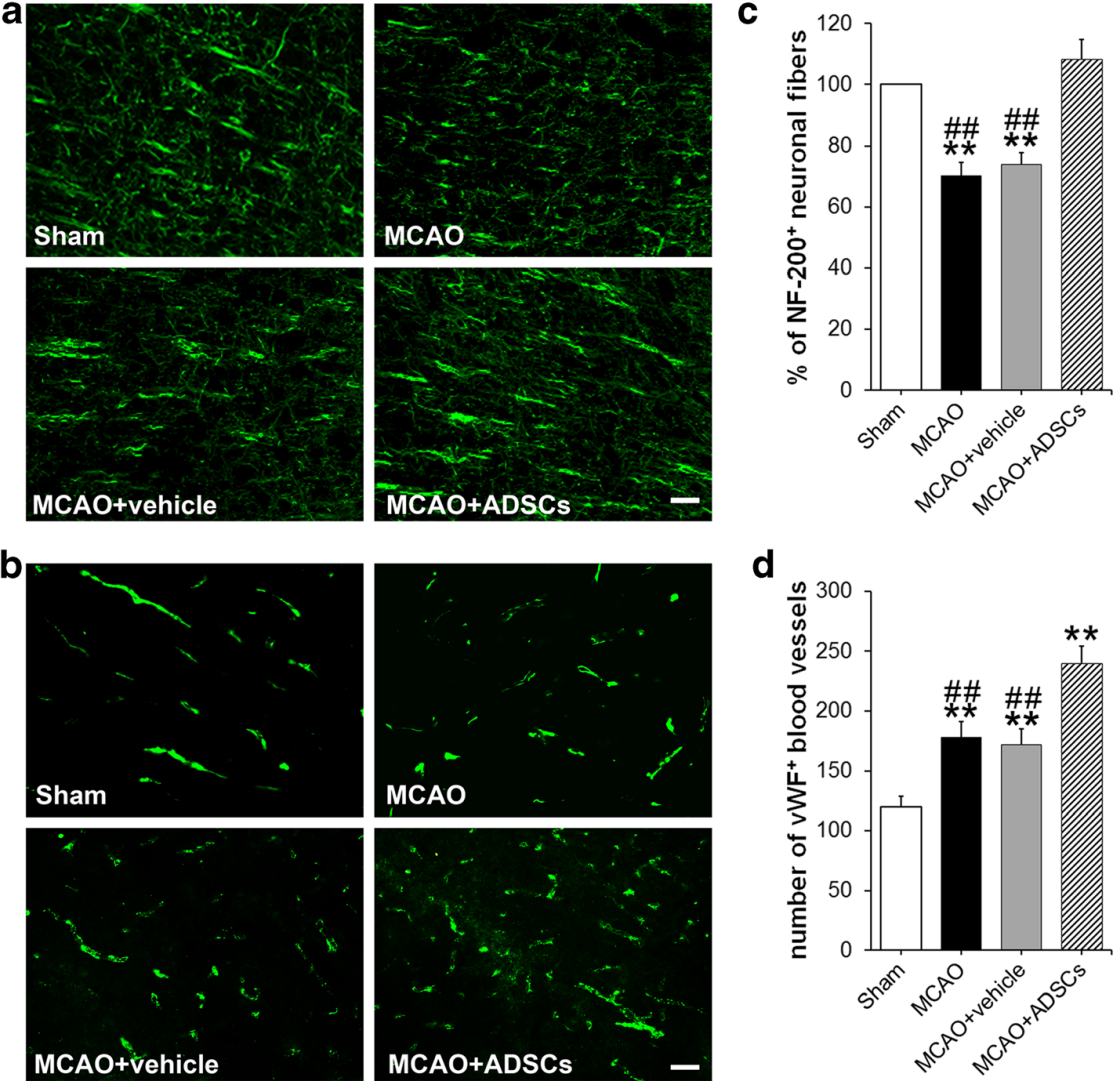
Regeneration of neuronal fibers and blood vessels. Immunofluorescence staining of the NF-200^+^ neuronal fibers (**a**) and vWF^+^ blood vessels (**b**) in the peri-infarct cortex of each group at day 14 after MCAO. *Scale bar* = 50 μm. Analysis of the integrated optical density of NF-200^+^ neuronal fibers (**c**) and number of vWF^+^ blood vessels (per mm^2^) (**d**) in each group. **p < 0.01, compared with the Sham group; ^##^ *p* < 0.01, compared with the MCAO + ADSC group. *ADSC* adipose-derived stem cell, *MCAO* middle cerebral artery occlusion, *NF-200* neurofilament 200, *vWF* von Willebrand factor

NF-200 is mainly expressed in neuronal fibers (including axons and dendrites). Previous studies have found that, compared with the sham-operated rats, NF-200^+^ neuronal fibers reduced to about 60–70% in the peri-infarct area at day 7 after MCAO, and then increased to about 80% and 100% of intact levels at days 28 and 56 after MCAO, respectively [32]. In this study, we measured the density of NF-200^+^ neuronal fibers in the peri-infarct cortex at day 14 after MCAO (Fig. 3a and c). Consistent with previous findings, the results showed that the density of NF-200^+^ neuronal fibers in the MCAO group and the MCAO + vehicle group significantly reduced to 70.21 ± 4.46% (*p* < 0.01) and 73.96 ± 3.72% (*p* < 0.01), respectively, compared with the Sham group (100 ± 0%). No significant difference was found between the Sham group and the MCAO + ADSC group (108.22 ± 6.54%, *p* > 0.05). Moreover, the density of NF-200^+^ neuronal fibers in the MCAO + ADSC group was respectively higher than that in the MCAO group and the MCAO + vehicle group (*p* < 0.01 for both), and no significant difference was evident between the MCAO group and the MCAO + vehicle group (*p* > 0.05), suggesting that ADSC transplantation accelerates the regeneration of neuronal fibers in the peri-infarct cortex of rats after MCAO.

### Regeneration of blood vessels in the peri-infarct cortex

vWF is a specific marker for endothelial cells, and evidence from animal models reveals that the number of vWF^+^ blood vessels increases in the peri-infarct area after stroke [30, 33]. In this study, we also counted the number of vWF^+^ blood vessels in the peri-infarct cortex at day 14 after MCAO (Fig. 3b and d). The results showed the number of vWF^+^ blood vessels was higher in the MCAO group, the MCAO + vehicle group, and the MCAO + ADSC group compared with the Sham group (178.19 ± 12.68 vs. 119.75 ± 9.03 per mm^2^, 171.88 ± 13.44 vs. 119.75 ± 9.03 per mm^2^, and 240.05 ± 14.29 vs. 119.75 ± 9.03 per mm^2^, respectively; *p* < 0.01 for all). Furthermore, the number of vWF^+^ blood vessels in the MCAO + ADSC group increased markedly when compared with that in the MCAO group and the MCAO + vehicle group (*p* < 0.01 for both), and no significant difference was found between the MCAO group and the MCAO + vehicle group (*p* > 0.05). These results suggest that ADSC transplantation enhances the angiogenesis in the peri-infarct cortex of rats after MCAO.

### Expression pattern of netrin-1 in the peri-infarct cortex

**Fig. 4:**
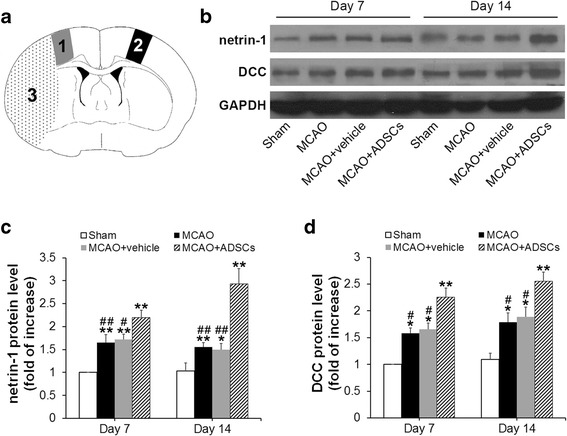
Western blot analysis of netrin-1 and DCC. **a** Schematic diagram of rat coronal brain section showing the peri-infract cortex (*1*), contralateral cerebral cortex (*2*) and infract core (*3*). **b** Western blot bands of netrin-1, DCC, and GAPDH in the peri-infarct cortex at days 7 and 14 after MCAO. Analysis of the expression levels of netrin-1 (**c**) and DCC (**d**). The band from day 7 in the Sham group is the calibrator sample representing the 1× expression. *p < 0.05, **p < 0.01, compared with the Sham group at the corresponding time points; ^#^ *p* < 0.05, ^##^ *p* < 0.01, compared with the MCAO + ADSC group at the corresponding time points. *ADSC* adipose-derived stem cell, *DCC* deleted in colorectal cancer, *MCAO* middle cerebral artery occlusion

To determine whether netrin-1 and DCC are involved in the neural protection induced by ADSC transplantation after ischemic stroke, we measured their temporal and spatial expression patterns in the brains of MCAO rats. Western blot analysis showed that the expression of netrin-1 in the MCAO group, the MCAO + vehicle group, and the MCAO + ADSC group increased at day 7 compared with the Sham group (1.65 ± 0.18 vs. 1 ± 0 fold, 1.72 ± 0.11 vs. 1 ± 0 fold, and 2.19 ± 0.16 vs. 1 ± 0 fold, respectively; *p* < 0.01 for all) and at day 14 (1.56 ± 0.09 vs. 1.03 ± 0.18 fold, *p* < 0.01; 1.49 ± 0.14 vs. 1.03 ± 0.18 fold, *p* < 0.05; and 2.93 ± 0.33 vs. 1.03 ± 0.18 fold, *p* < 0.01) (Fig. 4b and c). No significant difference in the expression level of netrin-1 was evident between the MCAO group and the MCAO + vehicle group at each time point (*p* > 0.05). However, ADSC transplantation significantly promoted netrin-1 expression in the MCAO + ADSC group when compared to that of the MCAO group and the MCAO + vehicle group at day 7 (*p* < 0.01 and *p* < 0.05, respectively) and day 14 (*p* < 0.01 for both).

**Fig. 5:**
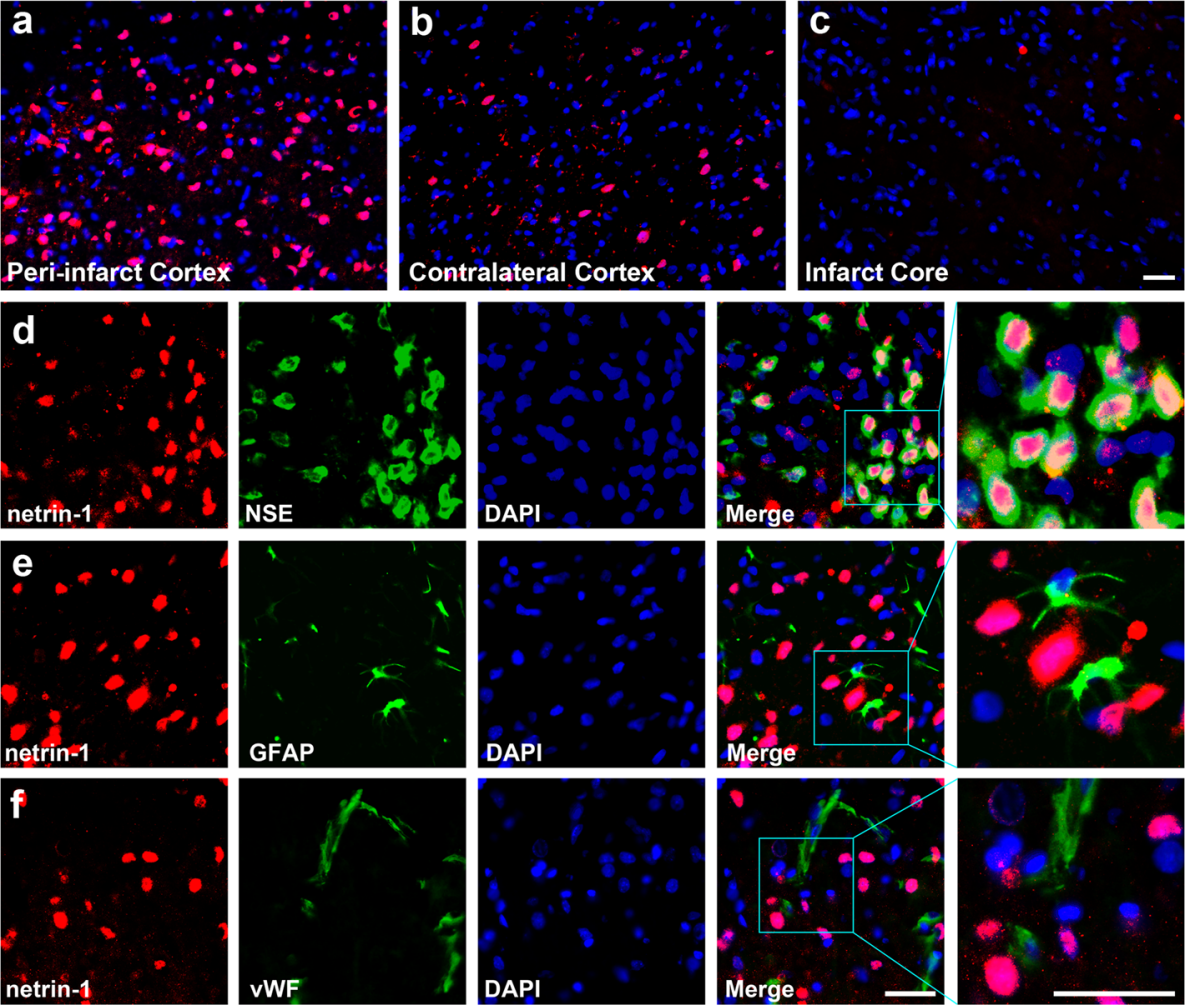
Immunofluorescence staining of netrin-1. The upper panels show the expression of netrin-1 in different areas of the brain at day 14 after MCAO. Netrin-1 was mainly expressed in the peri-infarct cortex (**a**), whereas weak immunoreactivity was found in the contralateral cerebral cortex (**b**), and no visible immunoreactivity was observed in the infarct core (**c**). The lower three panels show the dual-labeled immunofluorescence for netrin-1 (*red*) and neuron-specific enolase (*NSE*; *green*) (**d**), netrin-1 and glial fibrillary acidic protein (*GFAP*; *green*) (**e**), and netrin-1 and von Willebrand factor (*vWF*; *green*) (**f**) in the peri-infract cortex at day 14 after MCAO, respectively, and nuclei were stained with 4,6-diamidino-2-phenylindole (*DAPI*; *blue*). Netrin-1 was mainly expressed in NSE^+^ neuronal perikarya, but did not colocalize with GFAP^+^ astrocytes and vWF^+^ blood vessels. *Scale bar* = 50 μm

Immunofluorescence staining revealed that netrin-1 was mainly expressed in the peri-infarct cortex (Fig. 5a). Weak immunoreactivity was found in the contralateral cerebral cortex of the experimental groups and the area in sham-operated rat brains which corresponded to the peri-infarct cortex (Fig. 5b), whereas no visible immunoreactivity was observed in the infarct core (Fig. 5c). Dual-labeled immunofluorescence for netrin-1 and NSE or GFAP or vWF was performed to determine the phenotype of cells that expressed netrin-1. Consistent with previous studies [24, 25], we found that netrin-1 was mainly expressed by NSE^+^ neuronal perikarya (Fig. 5d), but did not colocalize with GFAP^+^ astrocytes (Fig. 5e) and vWF^+^ blood vessels (Fig. 5f). A previous study reported that netrin-1 was also detected in oligodendrocytes [24], but the current study found that netrin-1^+^ cells did not appear to be of the oligodendrocyte phenotype, which is consistent with the study of Liu et al. [25].

### Expression pattern of DCC in peri-infarct cortex

Western blot analysis showed that the expression level of DCC in the MCAO group, the MCAO + vehicle group, and the MCAO + ADSC group increased at day 7 compared with the Sham group (1.58 ± 0.10 vs. 1 ± 0 fold, *p* < 0.05; 1.66 ± 0.12 vs. 1 ± 0 fold, *p* < 0.05; and 2.26 ± 0.17 vs. 1 ± 0 fold, *p* < 0.01) and day 14 (1.79 ± 0.17 vs. 1.09 ± 0.12 fold, *p* < 0.05; 1.88 ± 0.19 vs. 1.09 ± 0.12 fold, *p* < 0.05; and 2.55 ± 0.17 vs. 1.09 ± 0.12 fold, *p* < 0.01) (Fig. 4b and b). No significant difference in the expression level of DCC was evident between the MCAO group and the MCAO + vehicle group at each time point (*p* > 0.05 for both). Meanwhile, the expression level of DCC in the MCAO + ADSC group was significantly higher than that in the MCAO group and the MCAO + vehicle group at each time point (*p* < 0.05 for both). Altogether, these findings reveal that ADSC transplantation induces higher expression of netrin-1 and DCC after MCAO.

**Fig. 6:**
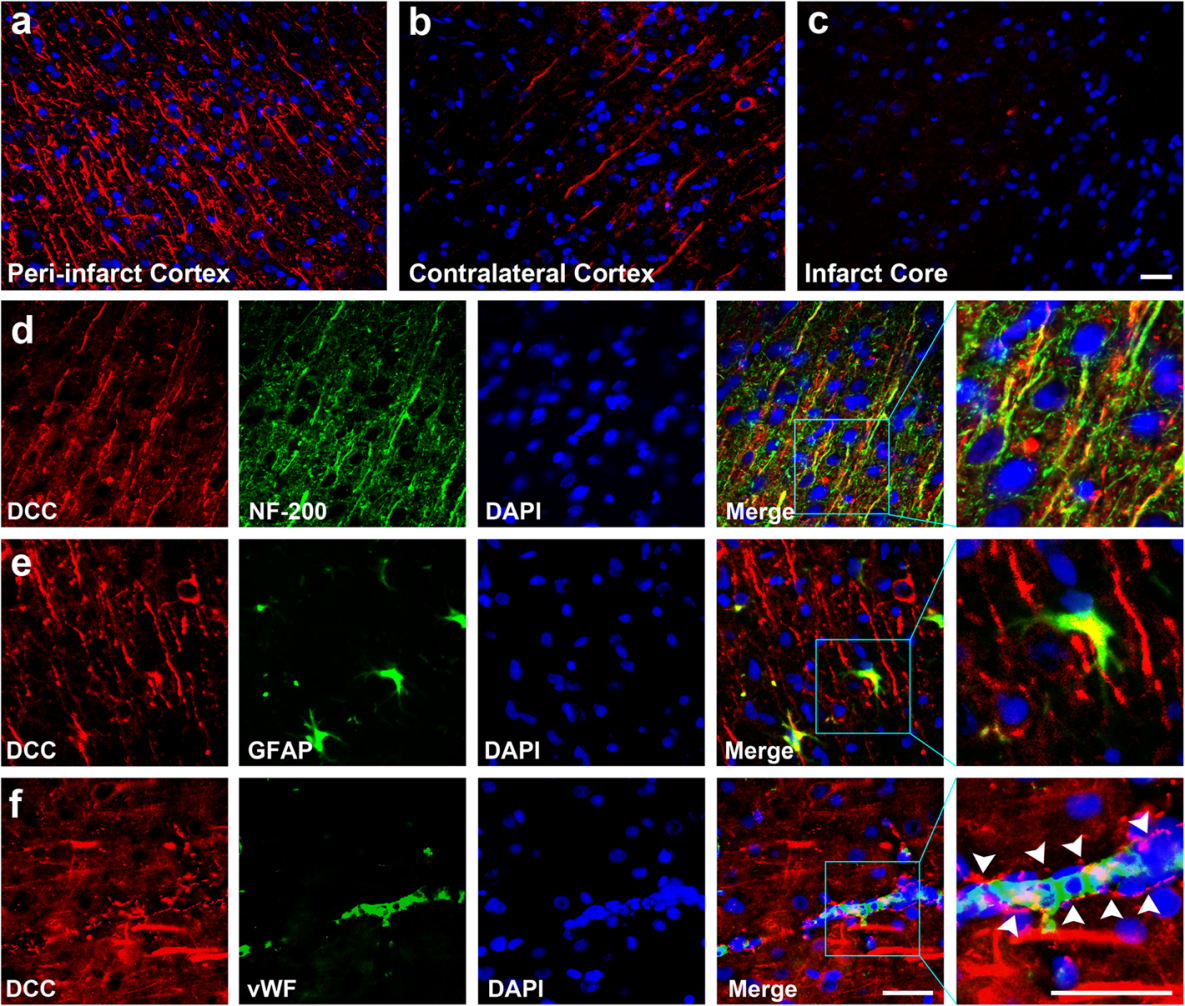
Immunofluorescence staining of DCC. The upper panels show the expression of deleted in colorectal cancer (*DCC*) in different areas of the brain at day 14 after MCAO. Immunoreactivity for DCC was predominantly found in the peri-infarct cortex (**a**), with little immunoreactivity in the contralateral cerebral cortex (**b**) and no visible immunoreactivity in the infarct core (**c**). The lower three panels show the dual-labeled immunofluorescence for DCC (*red*) and neurofilament-200 (*NF-20*0; *green*) (**d**), DCC and glial fibrillary acidic protein (*GFAP*; *green*) (**e**), and DCC and von Willebrand factor (*vW*F; *green*) (**f**) in the peri-infract cortex at day 14 after MCAO, respectively, and nuclei were stained with 4,6-diamidino-2-phenylindole (*DAPI*; *blue*). DCC mainly colocalized with NF-200^+^ neuronal fibers, and sparsely with GFAP^+^ astrocytes. Though DCC did not colocalize with vWF^+^ blood vessels, obvious DCC immunoreactivity was evident around the vWF^+^ blood vessels (*white arrowheads*). *Scale bar* = 50 μm

Immunoreactivity of DCC was similar to that of netrin-1, which was predominantly found in the peri-infarct cortex (Fig. 6a). Little immunoreactivity was found in the contralateral cerebral cortex of the experimental groups and the area in sham-operated rat brains which corresponded to the peri-infarct cortex (Fig. 6b). No visible immunoreactivity was observed in the infarct core (Fig. 6c). Dual-labeled immunofluorescence for DCC and NF-200 or GFAP or vWF was respectively performed. The results demonstrated that DCC mainly colocalized with NF-200^+^ neuronal fibers (Fig. 6d), and sparsely with GFAP^+^ astrocytes (Fig. 6e). Of interest, though DCC did not colocalize with vWF, obvious DCC immunoreactivity was found around the vWF^+^ blood vessels (Fig. 6f). On the basis of previous findings that DCC^+^ astrocytes wrap their end-feet around the blood vessels in the brain [25], and the current result that DCC colocalized with GFAP^+^ astrocytes in this study, we hypothesize that DCC wrapping around the blood vessels were expressed by the end-feet of the perivascular astrocytes.

## Discussion

The present study established a transient rat MCAO model receiving ADSC treatment and attempted to explore whether netrin-1 and DCC are involved in the neuroprotection of the stem cell-based therapy. The results showed that intra-arterially transplanted ADSCs effectively migrated toward the peri-infarct area and improved the neurological functions of MCAO rats. We also show that ADSC transplantation promoted the regeneration of neuronal fibers and blood vessels in the peri-infarct cortex, and that ADSC transplantation increased the level of netrin-1 and DCC protein in the peri-infarct cortex which were mainly expressed by neuronal perikaryal and fibers, respectively. These findings provide new clues about how ADSC transplantation promotes neurological recovery after stroke.

The available evidence from experimental animal models confirms that ADSC therapy can substantially improve neurological recovery after stroke [7, 10, 11, 34] indicating that this therapeutic scheme is a promising candidate for clinical application to stroke patients. Two important aspects need to be considered: the transplantation route of ADSCs and the optimal time point for ADSC therapy in acute ischemic stroke. As described in previous studies, a number of delivery routes for stem cells, including intra-arterial, intravenous, intraventricular, intracerebral, and intrastriatal, have been documented in experimental stroke models [4]. Du et al. [11] compared the therapeutic effects of the three most commonly used transplantation routes of ADSCs (intra-arterial, intravenous, and intraventricular) and found that, compared with intraventricular transplantation, intra-arterial or intravenous transplantation allowed higher dose injections with fewer invasions and appeared to be optimal in terms of therapeutic efficacy, safety, and feasibility [11]. Otero-Ortega et al. found that, after intravenous transplantation of ADSCs, migration and implantation of ADSCs were observed not in the brain, but in the liver, lung, and spleen, suggesting that most cells may be captured by peripheral organs with this delivery route [35].

Although ADSC therapy has a wider therapeutic window, which ranges from 30 min up to 14 days after stroke, early administration of ADSCs can achieve an efficient result for neuronal protection and repair [4]. Many studies have reported satisfactory results when the ADSCs were administered at 24 h after the induction of ischemic stroke [11, 34, 36]. Moreover, intra-arterial transplantation of ADSCs at 24 h after stroke showed the highest engraftment rate in the damaged brain of stroke rats [31]. Accordingly, the current study adopted the intra-arterial transplantation of ADSCs to rats at 24 h after MCAO. Consistent with previous studies, we demonstrated that ADSC transplantation significantly improved the neurological recovery of MCAO rats. We also provide evidence that ADSCs can effectively migrate toward the peri-infarct area via intra-arterial transplantation. Nevertheless, ADSCs were minimally detectable in the infarct core and other brain areas, which is probably due to the adverse environmental conditions of the infarct core where ADSCs are incapable of surviving, and the intact blood–brain barrier of other brain areas which makes it difficult for ADSCs to cross. We also noticed that, compared with the condition at day 7, ADSCs did not show a significant migration in the brain at day 14. This observation may be attributed to the following two factors: the rich chemokines in the peri-infarct area during the acute ischemic phase, which facilitates the recruitment of stem cells to the injury site [37], and the insufficient observation time in the current study. It has been reported that ADSCs are distributed on both hemispheres of the brain 8 weeks after intracerebral transplantation which indicates their robust in vivo migration [7]. More recently, the advent of molecular imaging techniques provides new and better means for noninvasive, repeated, and quantitative tracking of stem cells after transplantation, which is very useful in detecting, localizing, and examining the stem cells in vivo at both molecular and cellular levels [38, 39]. Therefore, molecular imaging techniques will help us better understand the behavior of stem cells and remedy the deficiency of this study.

Previous studies have demonstrated the beneficial effects of stem cell therapy on improving functional outcome after stroke through mechanisms implicated in brain plasticity, such as axonal sprouting, synaptic plasticity, remyelination, angiogenesis, and so on [4, 40]. In this study, we show that ADSC transplantation accelerated the regeneration of neuronal fibers and blood vessels in the peri-infarct cortex. It is generally believed that these plastic processes are stimulated by tropic factors (including brain-derived neurotrophic factor, vascular endothelial growth factor, hepatocyte growth factor, etc.) and other proteins (including synaptophysin, oligodendrocyte 2, microtubule associated protein 2, etc.) which are released or regulated by the ADSCs [4, 5, 9, 41], but the underlying molecular mechanisms remain only partially understood. In view of the facts that netrin-1 and DCC have also been found to be involved in these plastic processes [23], and that the overexpression of netrin-1 improves the functional recovery and reduces the infarct size in animal stroke models [42, 43], we measured the temporal and spatial expression patterns of netrin-1 and DCC in rat brains after ADSC transplantation. Western blot analysis revealed that the expression of netrin-1 and DCC increased in the peri-infarct cortex at days 7 and 14 after MCAO, which is consistent with results of previous studies [25]. We found that ADSC transplantation further enhanced the increased expression of netrin-1 and DCC at the same time point. Futhermore, immunofluorescence staining confirmed strong immunoreactivity for both netrin-1 and DCC in the peri-infarct cortex, but weak immunoreactivity in the contralateral cerebral cortex and no immunoreactivity in the infarct core, which bear great similarities to the locations of transplanted ADSCs in the rat brain. As the brain region adjacent to the infarct core, i.e., the peri-infarct area, is critical for the neurological rehabilitation due to its heightened neuroplasticity [44], the upregulation of netrin-1 and DCC in the peri-infarct cortex may reflect their potential role in the functional recovery induced by ADSC transplantation, discussed in greater detail below.

As we know, axonal injury and degeneration are prominent components resulting in reduced neuronal connectivity after acute ischemic stroke, which is largely responsible for the consequent impairment of neurological function [45]. Axonal regeneration and reorganization among surviving neurons take place simultaneously after stroke, trying to repair and re-establish lost connections, but this process is exceedingly difficult due to the limited neuronal intrinsic potential for axonal sprouting and regeneration in the central nervous system [45, 46]. Fortunately, netrin-1 and its receptor DCC have been well documented to not only enhance the capability of axons to sprout and regenerate, but also facilitate the generation of appropriate neural circuits by navigating these axons toward their ultimate targets, where synapses are formed [15, 21]. Moreover, netrin-1 has been found to affect dendritic outgrowth and targeting, and regulate synaptic function and plasticity in the adult mammalian brain via a DCC-dependent mechanism [47, 48]. In this study, the enhanced expression of netrin-1 and DCC was detected in the peri-ischemic cortex, and immunofluorescence staining showed that netrin-1 was expressed mainly in the neuronal perikarya while DCC was expressed extensively in neuronal fibers of this area. These findings suggest that netrin-1 may be secreted by neurons and then triggers axonal regeneration and reorganization to restore neuronal circuits after the cerebral ischemia by binding to its receptor DCC expressed in neuronal fibers. Furthermore, ADSC transplantation may promote this neuronal network remodeling by upregulating their expression.

As the cross-talk between nervous and vascular systems has been documented to occur at the molecular level during brain development and injury, both systems not only are similar in their anatomical form and structure but also follow the same paths [49]. In view of this, studies have investigated the role of netrin-1 in the vascular system and reported that netrin-1 promotes both developmental and therapeutic angiogenesis due to its ability to regulate the proliferation, differentiation, migration, and survival of endothelial cells [50–52]. Fan et al. demonstrated that netrin-1 induced blood vessel formation in the brain of adult mice and ameliorated cerebrovascular development and remodeling [52]. Other studies have reported that netrin-1 accelerated revascularization and reperfusion in mice suffering from hindlimb ischemia [20, 53], and that netrin-1 augmented the angiogenesis of mesenchymal stem cells and improved the function of the ischemic hindlimb [51]. Also, netrin-1 is found to be superior to vascular endothelial growth factor in restoring nerve conduction velocity, possibly due to its strong biological effects on both the nervous and vascular system [20]. Thus, the beneficial effects of ADSC transplantation on promoting angiogenesis after stroke may involve netrin-1 in the repair and re-establishment of the vascular network in the peri-infarct cortex. In this study, we did not detect DCC immunoreactivity in blood vessels, suggesting that the proangiogenic effect of netrin-1 in the brain might act through other receptors; this awaits further study. Nevertheless, it is noteworthy that obvious DCC immunoreactivity was observed to wrap around the blood vessels, which can be attributed to the end-feet of the perivascular astrocytes. Perivascular astrocytes have been found to engage in promoting brain neovascularization, vessel differentiation, and stabilization [52, 54], suggesting that angiogenesis induced by netrin-1 may be mediated partly through the DCC-expressing perivascular astrocytes. Moreover, as perivascular astrocytes have a supportive role in the maintenance of the brain–blood barrier [55], and netrin-1 has been found to support blood–brain barrier integrity and protect the central nervous system against injury [56, 57], we speculate that netrin-1 and DCC may also play a positive role in the restoration of the brain–blood barrier after cerebral ischemia, which also needs further clarification.

## Conclusions

The current study reveals that the intra-arterial transplantation of ADSCs can significantly improve the neurological recovery in a rat model of transient MCAO, and that transplanted ADSCs can effectively migrate toward the peri-infarct area of rat brains and accelerate the regeneration of neuronal fibers and blood vessels. The beneficial effects of ADSC transplantation may be attributed, at least in part, to the involvement of upregulated netrin-1 and its receptor DCC in the remodeling of neuronal and vascular networks in the peri-infarct cortex.

## Abbreviations

ADSC: Adipose-derived stem cell
CCA: Common carotid artery
DAPI: 4,6-Diamidino-2-phenylindole
DCC: Deleted in colorectal cancer
ECA: External carotid artery
GFAP: Glial fibrillary acidic protein
ICA: Internal carotid artery
IgG: Immunoglobulin G
MCAO: Middle cerebral artery occlusion
mNSS: Modified neurologic severity scores
NF-200: Neurofilament-200
NSE: Neuron-specific enolase
PBS: Phosphate-buffered saline
vWF: von Willebrand factor
